# Parents’ knowledge and attitudes regarding transthoracic device closure of VSD in children: a cross-sectional study

**DOI:** 10.1186/s13019-020-01124-z

**Published:** 2020-05-07

**Authors:** Ze-Wei Lin, Shu-Ting Huang, Ning Xu, Hua Cao, Qiang Chen

**Affiliations:** 1 Department of Cardiac Surgery, Fujian Maternity and Child Health Hospital, Affiliated Hospital of Fujian Medical University, Fuzhou, China; 2 Department of Cardiovascular Surgery, Union Hospital, Fujian Medical University, Fuzhou, China

**Keywords:** Knowledge, Attitude, VSD, Transthoracic device closure

## Abstract

**Objectives:**

This study aimed to identify Chinese parents’ knowledge and attitudes toward transthoracic device closure of ventricular septal defect (VSD).

**Methods:**

This cross-sectional study collected data on a total of 203 Chinese parents of patients with VSD were included, and an author-designed three-page questionnaire was used.

**Results:**

A total of 73.9% of the parents had heard of transthoracic device closure of VSD; however, they lacked detailed knowledge. 88.2% parents expressed their willingness to undergo this procedure. Although there was no significant correlation between knowledge about the occluder material and acceptance of the method, knowledge of other information was significantly related to willingness to undergo the procedure. Some parents expressed some concerns and high expectations, but the postoperative risk reduced their desire for accepting the procedure. This study also found that most parents did not have a detailed understanding of such procedure.

**Conclusion:**

Parents of patients with VSD in China need continued education regarding transthoracic device closure of VSD, especially in terms of its benefits and limited postoperative complications. In addition, it is essential to reduce the cost of this procedure to promote its development and application.

## Background

Ventricular septal defect (VSD) is a common congenital heart disease [1]. Surgical repair with cardiopulmonary bypass (CPB) is the traditional treatment; however, this treatment is associated with physical and psychological trauma, especially for pediatric and female patients [2–4]. Transcatheter device closure of VSD is a minimally invasive alternative for some patients [5–7]. This method is still associated with some adverse events, including malignant arrhythmia, device embolism, vascular injury, etc. In addition, it is limited by vascular size, especially in young patients. In recent years, transthoracic device closure of VSD, which combines the advantages of the above two methods, has been widely used in China [8–11]. It avoids a larger incision, CPB and radiation exposure. In addition, it has a short operative path, is easy to learn and perform and is less expensive than the other procedures. Considering the large population and different education levels in China, the large number of children with VSD born every year, and the limited scale of application of the transthoracic approach in some areas, parents may have some difficulty determining which treatment to seek for their children. Although many studies regarding the efficacy and safety of the transthoracic approach have been published in recent years [8–11], no studies have focused on the parents’ understanding of and attitudes toward this approach. The purpose of this study was to investigate the knowledge and attitudes of Chinese parents regarding transthoracic device closure of VSD and to provide recommendations for this treatment in different regions.

## Materials and methods

This cross-sectional survey used self-administered questionnaires for data collection. The survey was designed by the researchers and uses simplified Chinese. The three-page questionnaire collects data in three areas: (1) necessary information regarding the state of the human population (7 items), (2) knowledge about transthoracic device closure of VSD (7 items), and (3) attitudes toward transthoracic device closure of VSD (6 items). There were three possible answer options for some questions (know/do not know/unsure); “do not know” and “unsure” responses were considered wrong. The remaining questions had multiple choice answer options.

A total of 210 parents of patients with VSD volunteered to participate in the study between January 2018 and June 2018. The participants were informed of the purpose and content of the study before participating in the study. All participants provided written informed consent and were told that their participation was entirely voluntary and that they had the right and freedom to withdraw for any reason. This document survey was completed at the time of the patient’s first visit (outpatient or ward). The questionnaires were self-administered and filled out individually by the participants under the strict supervision by the research team. If the participants were confused by any of the questions, the researchers were available to help by addressing the participants’ problems or translating the questions into the local language. After the questionnaires were completed, other researchers collected and analyzed the data separately.

The data were analyzed using SPSS version 25.0 (IBM, Armonk, New York). Univariate analysis was used to describe the frequency and percentage of categorical variables. Descriptive statistics were used to identify the participants’ demographic characteristics and their knowledge and attitudes toward transthoracic device closure of VSD. The Pearson test was used to compare the attitudes of the parents toward the procedure. A *p* value< 0.05 was defined as statistically significant.

## Results

A total of 210 parents of children with VSD participated in the study, and 203 completed the questionnaire. The demographic characteristics of the respondents are shown in Table 1. The average age of the 203 participants was 29.5 ± 3.3 years. Most participants (74.4%) had completed at least a middle school education; 42.9% had a bachelor’s degree or above, and 25.6% had an education level of junior high school or below. A total of 34.0% of the participants were white-collar workers and civil servants, 27.1% were professionals, 15.3% were unemployed, 13.3% were farmers and 10.3% had other occupations. Approximately 87.7% lived in cities, and the rest lived in rural areas. Most of the participants had a monthly income of 5001–10,000 RMB (renminbi, Chinese currency) (57.6%); 16.3% had an income of 10,001–20,000 RMB, 13.8% had an income of less than 5000 RMB, and 12.3% had an income of more than 20,000 RMB.

**Table 1:** Baseline socio-demographic characteristics of participants

Characteristics	***N*** = 203 (n%)
**Age Range (years)**	
**20–25**	87 (42.9)
**26–30**	76 (37.4)
**31–35**	29 (14.3)
**> 35**	11 (5.4)
**Marriage**	
**Get married**	197 (97.0)
**Divorced**	6 (3.0)
**Education**	
**Junior High School or below**	52 (25.6)
**Middle school**	64 (31.5)
**Bachelor Degree or above**	87 (42.9)
**Career**	
**White-collar workers and civil servants**	69 (34.0)
**Professional person**	55 (27.1)
**Unemployed**	31 (15.3)
**Farmers**	27 (13.3)
**Other**	21 (10.3)
**Residential**	
**City**	178 (87.7)
**Rural**	25 (12.3)
**Monthly income (RMB)**	
**< 5000**	28 (13.8)
**5001–10,000**	117 (57.6)
**10,001–20,000**	33 (16.3)
**> 20,000**	25 (12.3)

***RMB*** Renminbi, Chinese currency

Table 2 presents additional information regarding the respondents’ knowledge of the transthoracic device closure of VSD. Only 73.9% (*n* = 150) of the respondents knew about transthoracic device closure of VSD, and the rest (26.1%) had not heard of this procedure. Of the people who knew about this method, nearly 34.0% knew about the different treatments for VSD, and more than half (85.3%) of the respondents believed that transthoracic device closure of VSD was risky. More than half (57.3%) knew that this procedure could be guided by transesophageal or transthoracic echocardiography. Unfortunately, only 18.0, 18.7, and 29.3% of respondents correctly answered questions about indications and contraindications, occluder materials, and postoperative complications, respectively. These items were used to measure knowledge about the procedure.

**Table 2:** Knowledge of transthoracic device closure of VSD in participants

Item	n b = 150	Correct answer n c (%)
**Q1 Have you ever heard of transthoracic device closure of VSD?**		
**Know**	150	
**Don’t know**	42	
**Unclear**	11	
**Q2 What are the main methods for VSD?**		
**Traditional Open thoracic Surgery**	42	51 (34.0)
**transthoracic device closure**	33	
**Percutaneous device closure**	24	
**All of the above**	51	
**Q3 Do you know the indications and contraindications of** **transthoracic device closure of VSD?**		
**Know**	27	27 (18.0)
**Don’t know**	123	
**Q4 Do you think there is a risk of transthoracic device closure of VSD?**		
**There are risks**	128	128 (85.3)
**No risk**	16	
**Unclear**	7	
**Q5 Do you know what can be as guiding tool?**		
**Transesophageal echocardiography**	40	86 (57.3)
**Transthoracic echocardiography**	46	
**Radiation**	23	
**Unclear**	41	
**Q6 Do you know what the material of the occluder?**		
**Alloy**	28	28 (18.7)
**Medical rubber**	41	
**Ceramic**	33	
**Unclear**	48	
**Q7 Do you know the main postoperative complications of this procedure?**		
**Know**	44	44 (29.3)
**Don’t know**	106	

N: the total number of participants. nb: the number of participants who have heard of transthoracic device closure of VSD. nc: the number of participants who correctly answer the following

When the respondents were asked, “Do you think there are risks involved in transthoracic device closure of VSD?” and “Do you know what can be used as a guiding tool?”, those who lived in the city, had higher education levels, held professional jobs and had high incomes, including white-collar workers and professionals, had a higher correct response rate (*P* < 0.05). The proportion of correct answers to this question was not significantly different from that for the question “Do you know the indications and contraindications for transthoracic device closure of VSD?” As shown in Table 3, the higher the respondent’s level of education, the higher the percentage of correct answers to the following questions: “What are the main methods for VSD?” and “Do you know what material the occluder is made from?” The respondent’s place of residence and education level were closely related to the percentage of correct answers (*P* < 0.05). For “Do you know the main postoperative complications of this procedure?”, the respondents’ career and monthly income levels were closely related to the percentage of correct answers (*P* < 0.05).

**Table 3:** **Factors related to the knowledge of transthoracic device closure of VSD(*n*** **= 150)**

	Q2, n1 = 51	Q3, n2 = 27	Q4, n3 = 128	Q5, n4 = 86	Q6, n5 = 28	Q7, n6 = 44
**Education**						
**Junior High School or below**	6 (11.8%)	5 (18.5%)	27 (21.1%)	18 (20.9%)	3 (10.7%)	10 (22.7%)
**Middle school**	13 (25.5%)	7 (25.9%)	46 (35.9%)	24 (27.9%)	7 (25.0%)	14 (31.8%)
**Bachelor Degree or above**	32 (62.7%)	15 (55.6%)	55 (42.3%)	44 (51.2%)	18 (64.3%)	20 (45.5%)
**X**^**2**^	16.524	3.03	8.293	9.204	8.447	0.654
**P**	0.00026	0.21981	0.01582	0.01003	0.01465	0.72108
**Career**						
**Unemployed**	7 (13.7%)	3 (11.1%)	15 (11.7%)	10 (11.6%)	4 (14.3%)	3 (6.8%)
**White-collar workers and civil servants**	20 (39.2%)	11 (40.7%)	49 (38.3%)	36 (41.9%)	8 (28.6%)	26 (59.2%)
**Professional person**	18 (35.3%)	8 (29.6%)	38 (29.7%)	28 (32.5%)	10 (35.7%)	10 (22.7%)
**Farmers**	4 (7.8%)	3 (11,1%)	17 (13.3%)	9 (10.5%)	4 (14.3%)	3 (6.8%)
**Other**	2 (3.9%)	2 (7.4%)	9 (7.0%)	3 (3.5%)	2 (7.1%)	2 (4.5%)
**X**^**2**^	6.55	1.05	17.452	15.448	1.479	17.654
**P**	0.16163	0.90212	0.00158	0.00386	0.83035	0.00144
**Residential**						
**City**	44 (86.3%)	24 (88.9%)	120 (93.8%)	82 (95.3%)	26 (92.9%)	39 (88.6%)
**Rural**	7 (13.7%)	3 (11.1%)	8 (6.2%)	4 (4.7%)	2 (7.1%)	5 (11.4%)
**X**^**2**^	1.192	0.045	13.636	6.407	8.929	0.129
**P**	0.27493	0.832	0.00022	0.01137	0.00281	0.71947
**Monthly income (RMB)**						
**< 5000**	5 (9.8%)	2 (7.4%)	5 (3.9%)	4 (4.7%)	3 (10.7%)	4 (9.1%
**5000–1000**	30 (58.8%)	17 (63.0%)	90 (70.3%)	58 (67.4%)	14 (50.0%)	21 (47.7%)
**10,000–20,000**	9 (17.6%)	3 (11.1%)	18 (14.1%)	10 (11.6%)	4 (14.3%)	8 (18.2%)
**> 20,000**	7 (13.7%)	5 (18.5%)	15 (11.7%)	14 (16.3%)	6 (21.4%)	11 (25.0%)
**X**^**2**^	1.184	1.578	23.561	7.946	4.108	12.872
**P**	0.75684	0.66439	0.00003	0.04714	0.25004	0.00492

N is the total number of participants. n denotes the number of the participants who had heard of transthoracic device closure of VSD. n1,n2,n3,n4,n5,n6 denote the number of the participants who correctly answered the Q2, Q3, Q4, Q5, Q6, Q7, respectively. *RMB* Renminbi, Chinese currency

Most (88.2%) of the respondents indicated that they were willing for their child to undergo transthoracic device closure of VSD. In terms of reasons, 42.4% of the respondents believed that this procedure was trustworthy and that their child would benefit from it (82.1%). Other questions were answered positively as follows: “I’m worried about the long scar” (39.1%), “I’m afraid of the long hospitalization” (48.0%) and “I am not worried about the cost” (26.8%). A total of 11.8% of the respondents expressed unwillingness to undergo this procedure, and more than half (75.0%) said that they were worried about unique risks related to the method. More than one-third (41.7%) felt that this procedure had not been applied on a large enough scale. Approximately 25.0% of the parents thought that the procedure was not very trustworthy. As many as 16.7% of the parents commented that there was no difference between this procedure and traditional ones and that they could not accept the introduction of a foreign substance into their child’s body (Table 4).

**Table 4:** Reasons for rejection/acceptance of transthoracic device closure of VSD and the cost of expectation and acceptance

Variable	***N*** = 203 (n%)
**Have you ever heard of transthoracic device closure of VSD (yes)?**	150 (73.8)
**Would you like to take such procedure for your child?**	179 (88.2)
**Reasons for Acceptance**	*n* = 179
**The procedure is trustworthy**	76 (42.4)
**Patients will benefit from this procedure**	147 (82.1)
**I’m worried about the long scar**	70 (39.1)
**I’m afraid the long hospitalization**	86 (48.0)
**I’m not worried about the cost**	48 (26.8)
**Reasons for refusal**	*n* = 24
**It is not difference from the traditional one?**	4 (16.7)
**It hasn’t been popularized in large area scale**	10 (41.7)
**some unique risks related the procedure**	18 (75.0)
**This procedure is not very trustworthy**	6 (25.0)
**Unable to accept a foreign substance in child’s body**	4 (16.7)
**Expect**	
**High efficiency**	135 (66.5)
**More or less of a benefit**	73 (36.0)
**A little benefit**	9 (4.4)
**No benefit**	3 (1.5)
**Don’t know**	11 (4.8)
**Accepted costs (RMB)**	
**< 10,000**	85 (41.9)
**10,000–20,000**	80 (39.4)
**20,000–30,000**	11 (5.4)
**> 30,000**	27 (13.3)

***RMB*** Renminbi, Chinese currency

Another section in Table 4 shows the respondents’ expectations regarding transthoracic device closure for VSD. More than half (66.5%) of the respondents expected high benefits. Thirty-six percent of the respondents chose the option “some benefit”, and only 1.5% of the respondents thought that this procedure had no benefit. When asked about “accepted costs,” most of the respondents chose the lowest price option (< 10,000 RMB); 39.4% said they could accept a cost of 10,000–2000 RMB, while 5.4% thought they could pay 20,000–30,000 RMB. Only 13.3% of the respondents reported that they were willing to pay more than 30,000 RMB for the procedure.

Table 5 shows the factors that influenced the respondents’ opinions regarding accepting transthoracic device closure for VSD. In the univariate analysis, respondents with higher education levels and a higher monthly income were more likely to consent to the procedure (*P* < 0.001). In addition, participants living in the city and those engaged in professional work were more willing to agree to the procedure (*P* < 0.05). There was no significant correlation between knowing what material the occluder was made from and acceptance of the procedure (*P* > 0.05). The remaining items were significantly associated with the acceptance of transthoracic device closure of VSD.

**Table 5:** Factors related to make the decision

Socio-demographic statistics	Agree	Disagree	X^**2**^	P
**Education**				
**Junior High School or below**	41	16	20.166	0.00004
**Middle school**	58	4		
**Bachelor Degree or above**	80	4		
**Career**				
**Unemployed**	24	10	22.999	0.00013
**Farmers**	23	4		
**White-collar workers and civil servants**	64	2		
**Professional person**	51	2		
**Others**	17	6		
**Residential**				
**City**	161	14	17.784	0.00002
**Rural**	18	10		
**Monthly income (RMB)**				
**< 5000**	21	12	23.988	0.00003
**5001–10,000**	108	6		
**10,001–20,000**	29	4		
**> 20,000**	21	2		
**Knowledge of procedure**				
**Q2**				
**Correct**	50	1	8.006	0.00466
**Incorrect**	81	18		
**Q3**				
**Correct**	20	7	5.233	0.02216
**Incorrect**	111	12		
**Q4**				
**Correct**	118	10	18.589	0.00002
**Incorrect**	13	9		
**Q5**				
**Correct**	82	4	11.707	0.00062
**Incorrect**	49	15		
**Q6**				
**Correct**	22	6	2.389	0.12219
**Incorrect**	109	13		
**Q7**				
**Correct**	43	1	6.081	0.01366
**Incorrect**	88	18		

***RMB*** Renminbi, Chinese currency

## Discussion

VSD is a common congenital heart disease (CHD). Although it usually allows for a relatively long and active life, it often has a potential impact on lifestyle and life planning and requires some critical treatment decisions to be made [12–14]. Although a large number of studies on the efficacy and safety of transthoracic device closure of VSD have been published in recent years, the application and knowledge of such procedures is still relatively limited considering the large population in China [8–11]. Given that patients’ parents act as guardians, they should decide among the different treatments available for their children. The parents’ choice of treatment may have a significant impact on a patient’s future life, such as their longevity, pregnancy outcomes, career planning, and daily life, especially for younger patients [15–19].

No previous studies have focused on parents’ understanding of and attitudes toward transthoracic device closure of VSD. For parents to better understand, respond to and adhere to their children’s future lifestyle issues caused by heart defects and make treatments decisions based on their values and preferences, it is essential for them to be informed and involved in the decision-making process. We conducted this cross-sectional survey of parents of patients with VSD.

In our study, we selected parents of patients because they have the greatest understanding of the patient’s condition on which to base treatment decisions. Our study showed that nearly 73.9% of the respondents knew about transthoracic device closure of VSD. Recent efforts to increase knowledge of CHD may also increase awareness of VSD. The availability of relevant information varied greatly depending on the participants’ place of residence, sociodemographic factors, and educational level. Unfortunately, we found a lack of detailed knowledge regarding treatments among the respondents. Although more than half of the respondents had heard of transthoracic device closure of VSD, there was still a gap in their knowledge about indications, contraindications, occluder materials, and postoperative complications. Our survey also found that respondents from rural areas had a lower level of knowledge, but showed an overall high rate of acceptance of the transthoracic approach. In this study, the participants’ top three reasons for disapproving of this treatment were fear of risks related to the procedure, the feeling that the procedure has not been applied on a large enough scale and the feeling that the procedure is not trustworthy. Adequate education addressed specific knowledge gaps and common problems among parents in different regions, with different levels of education and from different cultures and could improve the acceptance of transthoracic device closure of VSD. However, it was unclear how institutions should best provide this education. In our study, differences in education level, occupation, area of residence, monthly income levels, and understanding of the treatment significantly affected the parents’ choices.

Our results also confirmed the importance of health education regarding the safety and efficacy of transthoracic device closure of VSD. Studies have shown that heart models created using 3D printing technology are useful for CHD education [20–22]. Such models inspired students’ interest in learning about CHD and improved the effectiveness of medical education. The 3D-printed heart models can represent real spatial relationships and permit the physical manipulation of external and intracardiac structures [20–22]. Therefore, we could use a heart model to make descriptions of this procedure more concrete, which would make parents would be more receptive to information about the procedure and more easily accepted it. Health care providers play an irreplaceable role in reducing the emotional concern of parents when their children undergo cardiac surgery. The low mortality rate and good long-term effects of corrective closure of VSD are now common, but the procedure may be costly for some patients with complex problems because they require longer hospitalizations and more care. The benefits of the transthoracic approach, such as the short operation time and the short hospitalization time, should be explained to families in detail so that they understand that this procedure will also reduce overall costs.

In addition, our study found that the participants hoped that the costs of the treatment would be less than 10,000 RMB. At present, the cost of surgical repair of VSD Is still higher than the cost of device closure of VSD in China due to the domestic occluder. Medical insurance in China is public and pays part of the medical expenses, so the patient’s parents still has to pay a small part of the expenses. These indicate that cost is a significant obstacle to the promotion of transthoracic device closure of VSD. The price should be set at an affordable level relative to its effectiveness. It is worth noting that we investigated the parents’ existing knowledge of transthoracic device closure of VSD to determine the specific gaps that exist in their acceptance of the approach and the type of related information can have the most significant impact on parents’ willingness for their children to receive this treatment.

## Limitations

The data were collected self-administered questionnaires, and some of the respondents may not have fully understood the issues presented, leading to potential bias. In China, many patients’ parents could get a certain amount of relevant knowledge through internet and WeChat platforms or books before they decided to see a doctor, and they might pay more attention to the contents of related diseases and inquire about the treatment status of other patients. These circumstances might also affect the conclusion. Unfortunately, we did not investigate the respondents’ preferences regarding the form of educational programs or where they were willing to have their child undergo the procedure. More importantly, this was a retrospective study with a certain amount of selective migration, and because no similar studies have been performed previously, vertical comparisons were not possible. Further research should be conducted to address these problems.

## Conclusion

In our study, although most of the respondents knew about transthoracic device closure of VSD, there was a lack of detailed understanding of relevant information. For the popularization of such treatment, the potential significance of our research is profound. Although the effect of transthoracic device closure of VSD is significant, to ensure the wide use of this procedure, promoters must increase awareness among populations in less developed areas; emphasize the safety, effectiveness, and risks of such treatment; and try to reduce the costs of the procedure.

## Data Availability

Data sharing not applicable to this article as no data sets were generated or analyzed during the current study.

## Abbreviations

VSD: Ventricular septal defect
CPB: Cardiopulmonary bypass
CHD: Congenital heart disease
